# Supplementary material for: Ultra-compact exciton polariton modulator based on van der Waals semiconductors
Source: Nat Commun. 2024 Mar 14;15:2331. doi: 10.1038/s41467-024-46701-1 (PMC10940672; doi:10.1038/s41467-024-46701-1)
Supplement: Supplementary file 1 — Supplementary Information [file 41467_2024_46701_MOESM1_ESM.pdf]

Supplementary Information for

Ultra-compact exciton polariton modulator based on van der Waals semiconductors

Seong Won Lee[1,2†], Jong Seok Lee[1,2†], Woo Hun Choi[1,2], Daegwang Choi[1,2], and Su-Hyun Gong[1,2*]

**Correspondence to:* shgong@korea.ac.kr

[†]*These authors contributed equally to this work*

Supplementary Note 1. The analytic calculation for guided polariton modulation

Supplementary Note 2. The coupled oscillator Hamiltonian model

Supplementary Figure 1. The detailed dimension of the fabricated polariton modulator

Supplementary Figure 2. E-k Dispersion Measurement using Fourier Microscope.

Supplementary Figure 3. Reconstruction of E-k Dispersion of guided exciton polariton in WS_2 waveguide.

Supplementary Figure 4. Power-dependent spectra measured at the excitation spot.

Supplementary Figure 5. The effect of polariton photoluminescence induced by a modulation laser.

Supplementary Figure 6. Comparison between the spectra of white light laser and the guided polariton signal.

Supplementary Figure 7. Modulation modeling based on the effective refractive index of guided polariton modes.

Supplementary Figure 8. Estimated polariton dispersion curves as a function of modulation laser power.

Supplementary Figure 9. Estimated photon and exciton fractions of guided polariton as a function of polariton energy.

Supplementary Figure 10. Control experiment for polariton modulation with a 685 nm modulation laser.

Supplementary Figure 11. Calculated propagation loss of guided exciton polariton mode in WS2 waveguide.

Supplementary Note 1. The analytic calculation for guided polariton modulation

In order to estimate the intensity of guided polaritons at the ends of the modulator, we employed a simplified model. As depicted in Fig. 1a, the WS_2 modulator is configured with two distinct paths, along arm 1 and arm 2. The focused modulation laser on arm 1 changes the effective refractive index of the guided exciton-polariton modes.

For the sake of simplicity, we assumed that the path lengths along arm 1 and arm 2 are equal, and the beam splitters within the modulator evenly distribute the guided modes in 50:50 ratios. Additionally, we made the assumption that the dimensions of the WS_2 waveguide in the modulator, including its thickness and width, remain uniform across the entire region. This uniformity ensures that the effective refractive index of the guided modes remains constant, except in the presence of the modulation laser.

The effective refractive index of the guided polariton mode without and with the modulation laser are represented as $n^* = n + ik$ and $n_m^* = n_m + ik_m$. It is important to note that both the real (n) and imaginary parts (k) of the effective refractive index have been considered.

In the absence of the modulation laser, the optical path length for the polariton flow in two paths is given by $n^* L$, where L is the total length of the modulator path. When the modulation laser is focused in arm 1 with a modulation length of L_m, the optical path length in one path is altered by $n^*(L - L_m) + n_m^* L_m$.

Consequently, the modulation ratio (I_m/I_o) is calculated using the equation:

$$\frac{I_m}{I_o} = \frac{\left| Ae^{i\frac{2\pi}{\lambda}[n^*(L-L_m)+n_m^* L_m]} + Ae^{i\frac{2\pi}{\lambda}n^* L} \right|^2}{\left| 2Ae^{i\frac{2\pi}{\lambda}n^* L} \right|^2}$$

where I_m and I_o are the intensities of the transmitted polariton with and without the presence of the modulation laser, respectively.

In order to dissect the impact of amplitude and phase modulation distinctly, we conducted calculations by intentionally deactivating the alteration of either the real or imaginary component of the effective refractive index. As depicted in Supplementary Figure 5, within shorter wavelength ranges, amplitude modulation is dominant owing to substantial shifts in the imaginary component of the effective refractive index. Conversely, in the longer wavelength region, the dominance of phase modulation effects (involving alterations in the real component of the effective refractive index) becomes evident. These calculation outcomes agree well with our control experimental findings (Fig. 2a-b).

Supplementary Note 2. The coupled oscillator Hamiltonian model

We utilize a simple coupled oscillator Hamiltonian model to calculate the exciton and photon fractions of guided polaritons as a function of polariton energy. The coupled oscillator Hamiltonian of exciton polaritons is

$$\begin{pmatrix} \hbar v q & \hbar\Omega_{Rabi}/2 \\ \hbar\Omega_{Rabi}/2 & E_{ex} \end{pmatrix}\begin{pmatrix} \alpha \\ \beta \end{pmatrix} = E_{pol}\begin{pmatrix} \alpha \\ \beta \end{pmatrix}$$

where E_{ex} is the exciton energy, $\hbar v q$ is the photon energy, and $\hbar\Omega_{Rabi}$ is the Rabi splitting energy. v is the phase velocity of photon $v = c/n_b$ where n_b is the background refractive index without considering exciton resonance, and E_{pol} represents upper and lower polariton energy, $E_{pol} = \frac{\hbar v q + E_{ex}}{2} \pm \frac{\sqrt{(\hbar\Omega_{Rabi})^2 + (\hbar v q - E_{ex})^2}}{2}$. α and β are the Hopfield coefficients, which was estimated by fitting the upper and lower polariton energy to the guided polariton dispersion curve in WS_2 waveguide (**S**upplementary Figure 9).

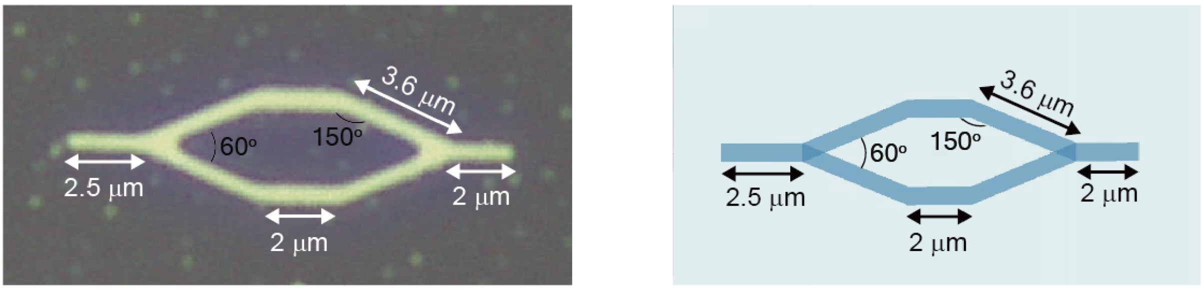

Supplementary Figure 1 | The detailed dimension of the fabricated polariton modulator. Here we employed a straightforward sample design as a proof-of-concept. However, optimizing the design of the MZI is important for enhancing the device's efficiency. Our initial proof-of-concept design exhibited some partial reflection at the beam splitter region. Refinements such as optimizing the angle of the beam splitter or incorporating a rounded bending of the polariton waveguide would reduce unnecessary losses arising from reflection and far-field radiation. Furthermore, we observed that the width of the waveguide plays a crucial role in MZI design. A wider waveguide induces spatial oscillations of the polariton fields in one arm due to the beating between different polariton modes. This spatial oscillation results in reduced overlap of the two polariton flows after the second beam splitter, thereby diminishing the interference effect. To mitigate this, we identified that maintaining the width of the polariton waveguide below 450 nm is essential to minimize the spatial beating effect

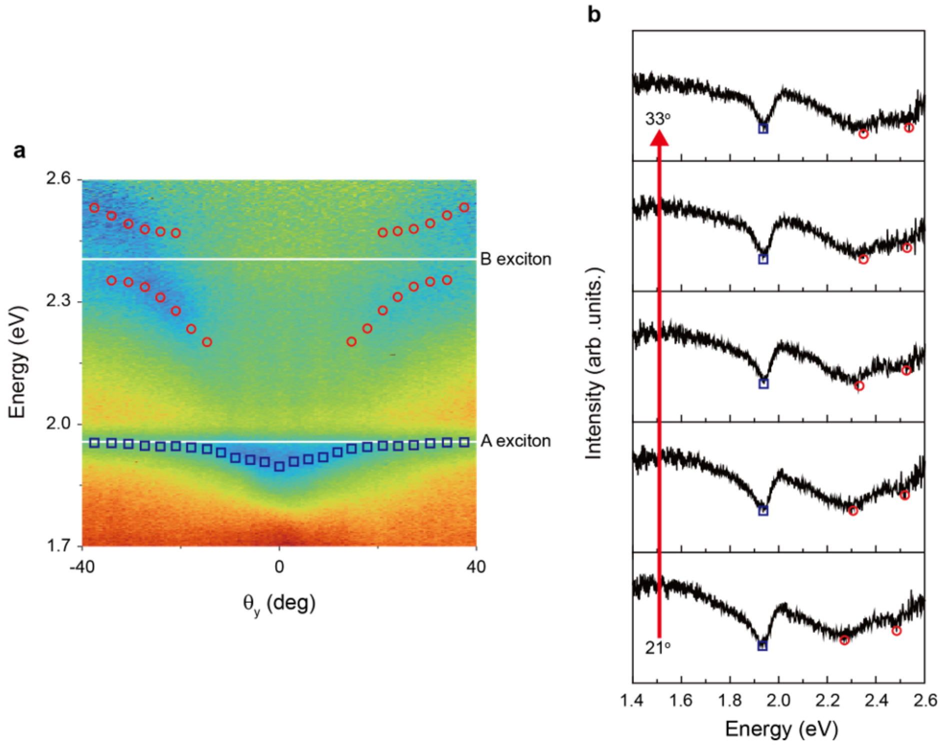

Supplementary Figure 2 | E-k Dispersion Measurement using Fourier Microscope. a, Measured angle-resolved reflection spectrum from grating pattered WS_2 waveguide. To demonstrate the presence of exciton-polariton mode in our waveguide, we employed a period structure to fold the E-k dispersion curve of the guided exciton-polariton into the air-light cone. The period and filling factor of the 1D grating structure were set to 480 nm and 0.42, respectively. Due to the very small width of the WS_2 waveguide (~480 nm), the signal-to-noise ratio of the data is very low. Nevertheless, the anti-crossing behavior of the polariton modes is visible near the exciton resonances. It is also important to point out that, even at the B exciton energy, which has smaller oscillator strength then A exciton, anti-crossing behavior is noticeable. **b,** Cross-section of the spectrum as a function of measured photon's angle. These results successfully indicate the presence of exciton-polariton modes in the WS_2 waveguide in the MZ modulator.

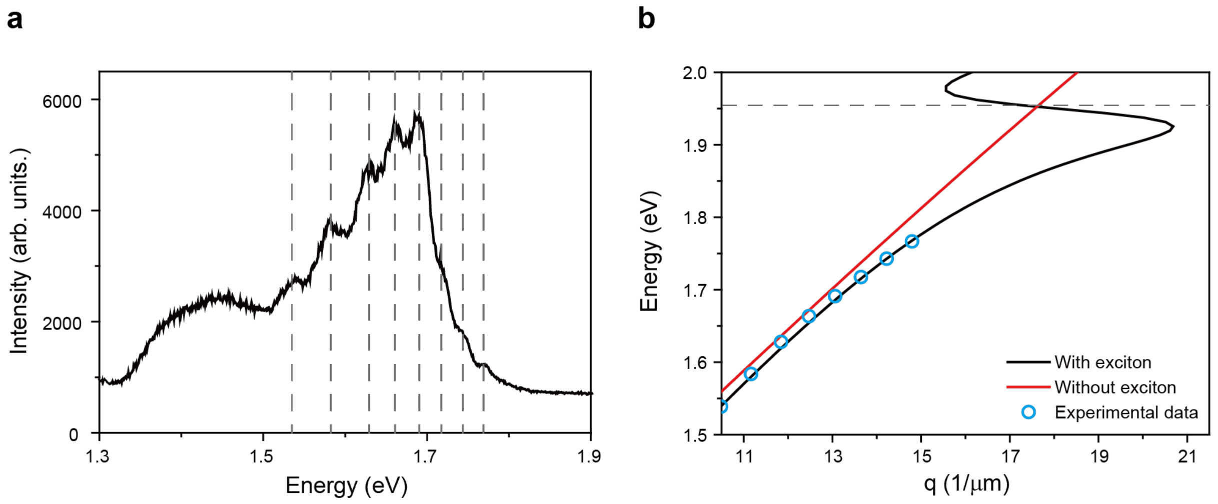

Supplementary Figure 3 | Reconstruction of E-k Dispersion of guided exciton polariton in WS_2 waveguide. a, Measured polariton spectrum scattered at the edge of the WS_2 waveguide. We attempted to measure the E-k dispersion of the guided polariton itself without folding it into the air light cone. We investigated interference peaks of guided-polariton spectrum, which is a typical method for estimating dispersion relations of a guided polariton along a waveguide[1]. Because the spectral distribution of interference peaks is strongly related to lateral momentum (q) as a function of frequency, we could directly estimate the E-k dispersion relation of guided modes from the interference peaks measured at a sample edge. **b,** The distribution of the interference peaks (solid circles) was fitted on the calculated dispersion curves (solid lines). The interference peaks were placed in lateral momentum (q) at $q=2\pi(m/L+1/\lambda)$. We considered the interference between the polariton path and the direct reflection of the white light laser from the glass substrate. L and m are a path difference and an integer number for the interference peaks. Thus, gradually decreasing energy spacing between adjacent interference peaks near the exciton energy (~1.97 eV) directly indicates the dispersion curve should be curved near the exciton resonance rather than a straight light line. These experimental data successfully support the existence of exciton polariton modes.

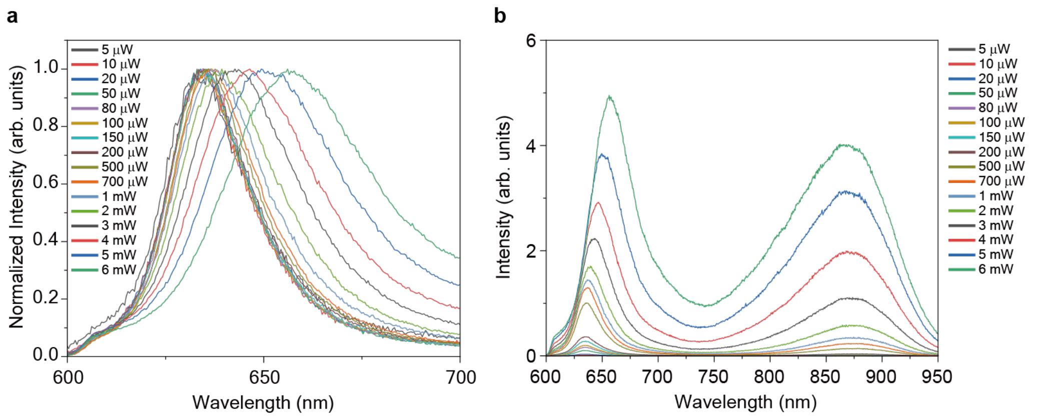

Supplementary Figure 4 | Power-dependent spectra measured at the excitation spot. a, The normalized exciton spectrum of WS_2 multilayers under varying pumping power of the 514 nm continuous-wave laser. **b,** The whole measured spectrum of WS_2 multilayers. The wavelength of indirect emission (~870 nm) was not strongly influenced by the modulation laser power.

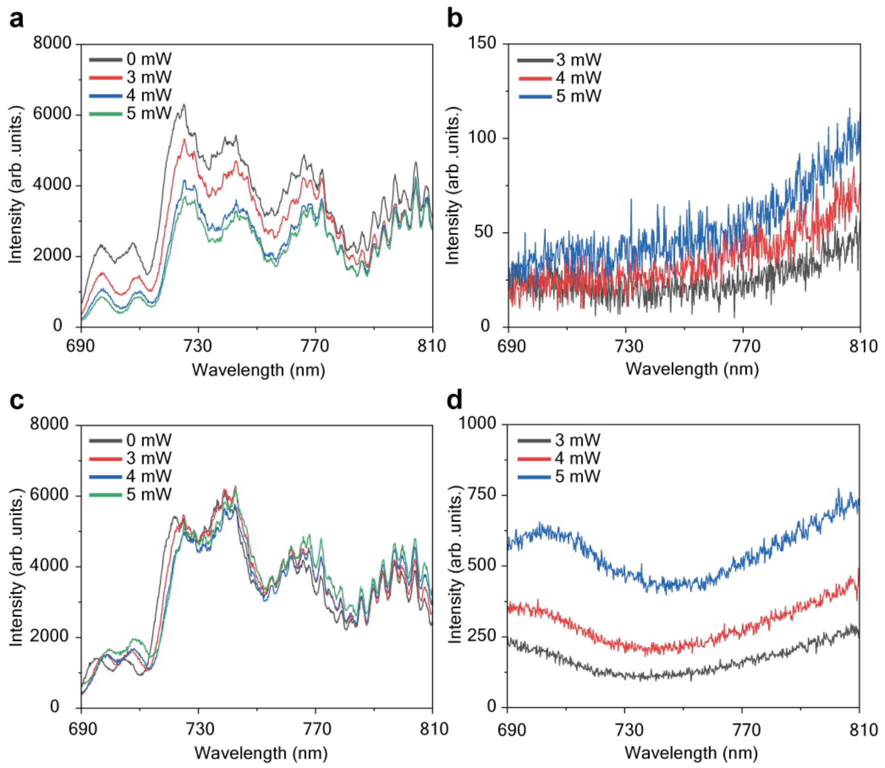

Supplementary Figure 5 | The effect of polariton photoluminescence induced by a modulation laser. a,b Guided polariton spectrum with a focused modulation laser on one arm of the MZ modulator (see Fig. 2a in the main text). **a,** Guided polariton spectrum measured at the edge of the WS_2 modulator with different modulator laser powers. **b,** Guided polariton spectrum as a function of modulator pumping power without the presence of resonantly excited polariton by the white light laser. The nonresonantly excited polariton signal by the modulation laser is negligible compared to the intensity of resonantly excited polariton. **c,d,** Guided polariton spectrum with a focused modulation laser after a second beam splitter of the MZ modulator (see Fig. 2b in the main text). **c,** Guided polariton spectrum with different modulator laser powers. **d,** Guided polariton spectrum without the presence of resonantly excited polariton by the white light laser. The photoluminescence (PL) intensity is higher than the spectrum in **b** due to closer excitation position to the edge, however, the signal is still negligible.

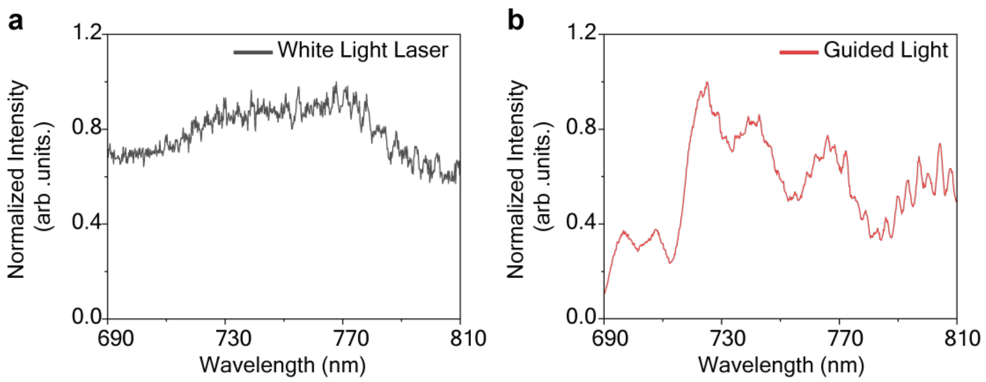

Supplementary Figure 6 | Comparison between the spectra of white light laser and the guided polariton signal. a, Spectrum of white light laser reflected from the glass substrate. **b,** Spectrum of guided light along the WS_2 modulator.

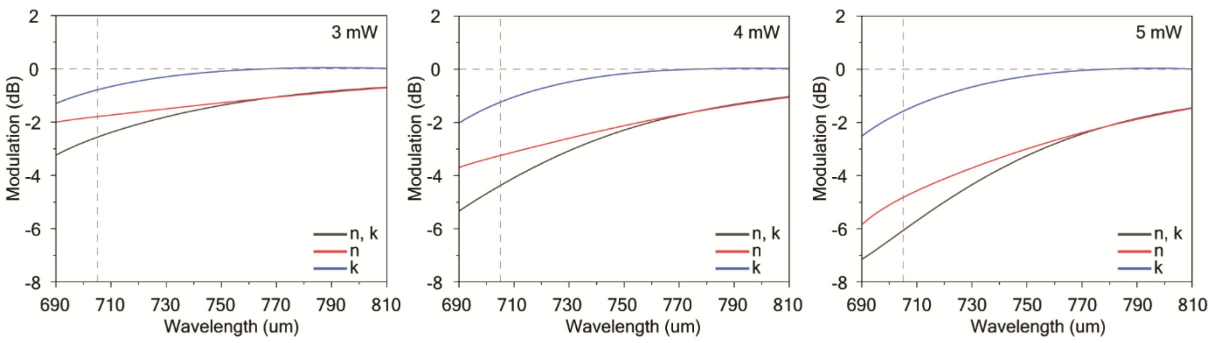

Supplementary Figure 7 | Modulation modeling based on the effective refractive index of guided polariton modes. Refer to Supplementary Note 1 for the detailed calculation methodology. Modulation intensity ratios were calculated by accounting for alterations in both the real (n) and imaginary (k) components of the effective refractive index upon modulation laser pumping. Additionally, modulation intensities were calculated while intentionally disregarding changes in other components of the effective refractive index.

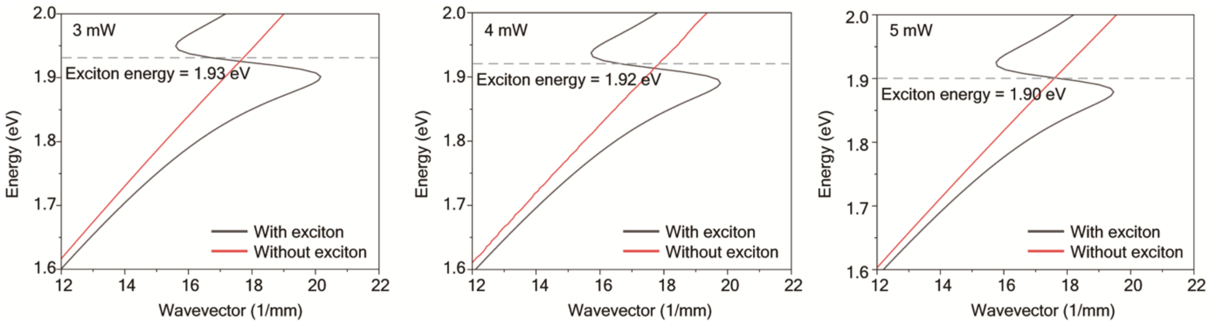

Supplementary Figure 8 | Estimated polariton dispersion curves as a function of modulation laser power. Polariton dispersion curves were calculated based on the refractive index of WS_2 presented in Fig. 3a.

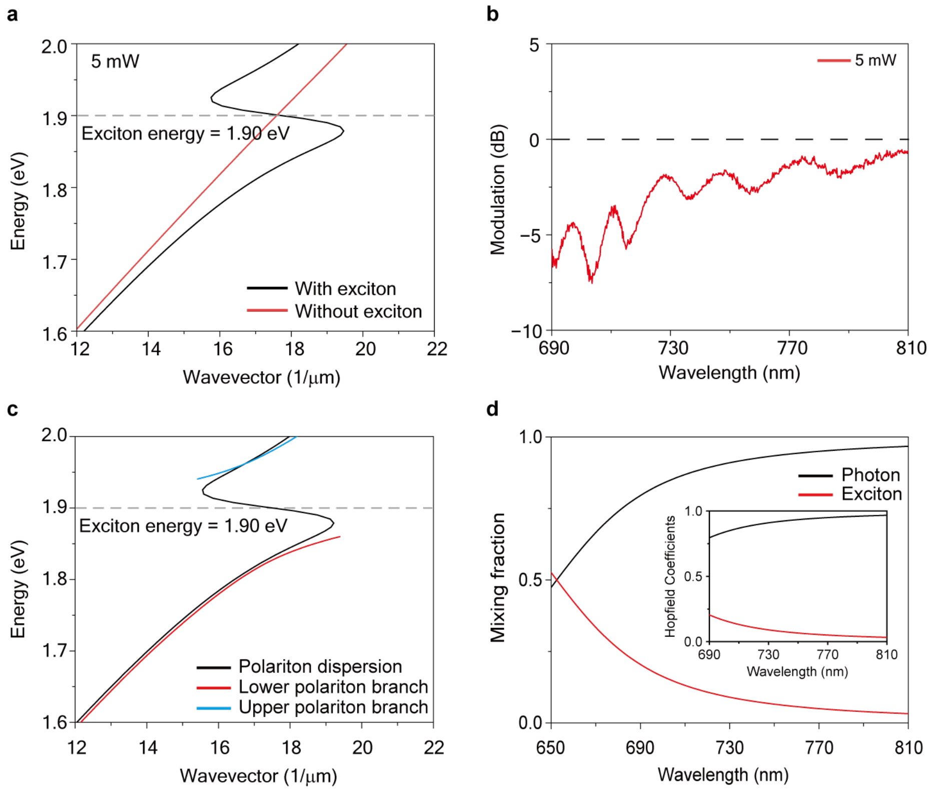

Supplementary Figure 9 | Estimated photon and exciton fractions of guided polariton as a function of polariton energy. a, Calculated dispersion relation of the guided mode in a WS_2 waveguide using semi-classical model (i.e., Lorenz oscillator model) **b**, Experimentally measured modulation ratio as a function of wavelength **c**, Calculated dispersion relation of upper and lower polariton using coupled oscillator Hamiltonian model (See Supplementary Information for details). A small mismatch near the exciton resonance is due to the neglect of exciton loss in the Hamiltonian model. **d**, The mixing fraction of photon and exciton as a function of wavelength, estimated using the Hopfield coefficient of the Hamiltonian model. The results directly demonstrate that the exciton fraction decreases as the polariton energy moves away from the exciton energy. When comparing the mixing fraction results with the modulation efficiency as a function of wavelength, we observe that a higher exciton fraction results in more efficient modulation.

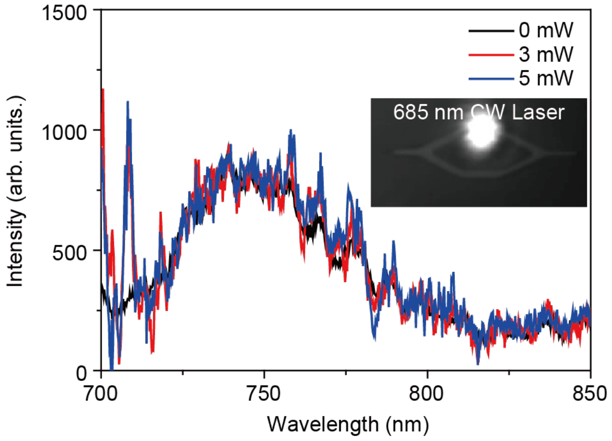

Supplementary Figure 10 | Control experiment for polariton modulation with a 685 nm modulation laser. To clarify the significance of the modulation laser wavelength, we conducted a control experiment with a 680 nm modulation laser. Focusing the 680 nm laser on one arm of the MZI, where excitons cannot be excited, resulted in no change in polariton transmission. While sharp noisy peaks appear due to the laser tails, the overall shape of the polariton spectrum was not influenced by the 680 nm modulation laser. This result demonstrates that exciton excitation is crucial for inducing modulation. Firstly, the laser with exciton excitation can induce a higher crystal temperature change owing to the thermal relaxation processes of excitons. Secondly, high exciton density also causes a bandgap renormalization effect resulting in the red-shift of the exciton energy. Therefore, the resultant redshift of exciton energy under exciton pumping leads to an effective refractive index change of the guided exciton polariton.

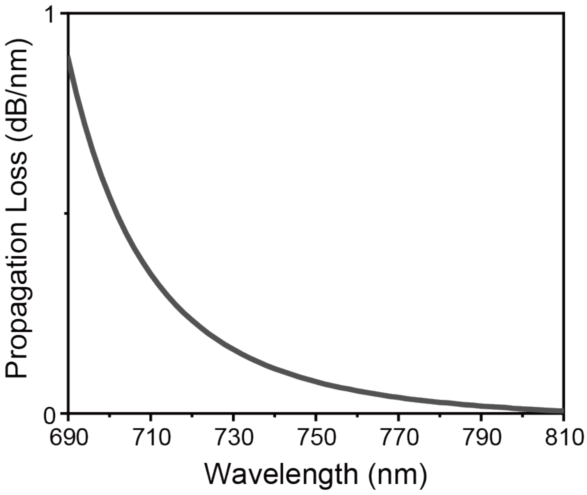

Supplementary Figure 11 | Calculated propagation loss of guided exciton polariton mode in WS_2 waveguide.

Supplementary References

1 Van Vugt, LK. *et al.* One-dimensional polaritons with size-tunable and enhanced coupling strengths in semiconductor nanowires. *PNAS* **108**, 10050-10055 (2011)
